# Electrofreezing of Liquid Ammonia

**DOI:** 10.1021/acs.jpclett.2c02576

**Published:** 2022-10-18

**Authors:** Giuseppe Cassone, Jiri Sponer, Judit E. Sponer, Franz Saija

**Affiliations:** † Institute for Chemical-Physical Processes, National Research Council of Italy, Viale F. Stagno d’Alcontres 37, 98158 Messina, Italy; ‡ Institute of Biophysics of the Czech Academy of Sciences, Královopolská 135, 61265 Brno, Czechia; ¶ Regional Center of Advanced Technologies and Materials, The Czech Advanced Technology and Research Institute (CATRIN), Palacky University Olomouc, Slechtitelu 27, 77900 Olomouc, Czechia

## Abstract

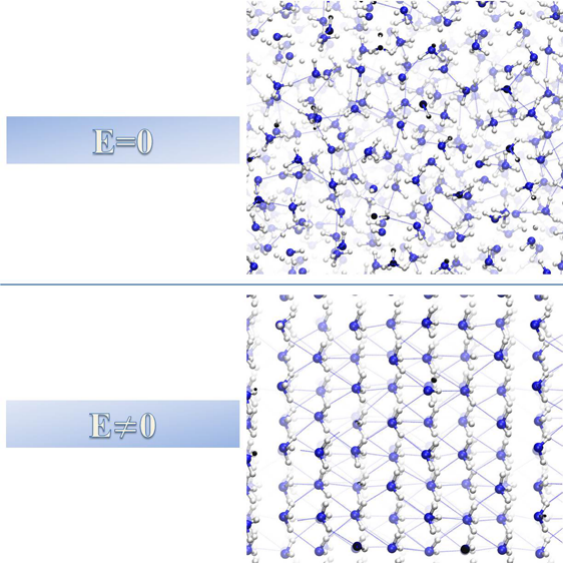

Here we prove that, in addition to temperature and pressure, another important thermodynamic variable permits the exploration of the phase diagram of ammonia: the electric field. By means of (path integral) *ab initio* molecular dynamics simulations, we predict that, upon applying intense electric fields on ammonia, the electrofreezing phenomenon occurs, leading the liquid toward a novel ferroelectric solid phase. This study proves that electric fields can generally be exploited as the access key to otherwise-unreachable regions in phase diagrams, unveiling the existence of new condensed-phase structures. Furthermore, the reported findings have manifold practical implications, from the safe storage and transportation of ammonia to the understanding of the solid structures this compound forms in planetary contexts.

Ammonia (NH_3_), owing to its ubiquity and apparent simplicity, is among the most employed molecular species. Such a compound covers, indeed, a central role in the manufacture of fertilizers, explosives, and pharmaceuticals.^1^ Beyond its terrestrial relevance, ammonia plays a key geophysical role in a plethora of planetary contexts. In fact, NH_3_ is present in the atmospheres of Jupiter and Saturn^2^ and in the mantle of the ice giants Uranus^3^ and Neptune.^4^ As a result of its abundance at the isobars where Jovian lightning is produced, ammonia is believed to hold a pivotal place in the realization of electrical storms on Jovian-like planets.^5^ In addition, detection of ammonia in super-Earth exoplanets has also largely been accredited.^6^

Similarly to water, ammonia exhibits complex—but weaker—three-dimensional and dynamical hydrogen-bonded (H-bonded) networks in the condensed phase. Depending on temperature and pressure conditions, different solid phases of ammonia are realized.^7−9^ Below ∼4 GPa, the NH_3_ phase diagram displays three solid phases, having structures which are pseudoclose-packed with ordered molecules (phase I) or genuinely close-packed with orientationally disordered molecules, such as phase II (hexagonal-close packed, *hcp*) and phase III (face-centered cubic, *fcc*).^8^ At higher pressures all these structures undergo a first-order phase transition toward phase IV, an orthorhombic crystal with pseudo-*hcp* packing.^9^ Only at 14 GPa does phase IV evolve into phase V, another orthorhombic structure. As predicted by early *ab initio* molecular dynamics (AIMD) simulations,^10^ at high temperatures (>700 K) and pressures (>57 GPa), ammonia undergoes molecular dissociation and a superionic solid phase α is found, where the crystal structure is made of the ammonia counterions (NH_4_^+^ and NH_2_^–^) while protons flow across the nitrogen sublattice with large mobility.^11^ Furthermore, it was forecasted that at low temperatures and above ∼90 GPa ammonia evolves into a crystalline ionic solid phase consisting of alternate layers of NH_4_^+^ and NH_2_^–^ ions.^12^ A synergistic combination of experiments and *ab initio* calculations later confirmed the existence of those structures.^13^

Electrofreezing (i.e., the electric-field-induced crystallization) is known to be relevant in many natural processes, ranging from tropospheric dynamics to frost damage in cells.^14−17^ Albeit historical molecular dynamics simulations predicted the possibility of freezing (supercooled) liquid water by means of static electric fields (EFs),^18,19^ recent laboratory experiments,^20^ AIMD,^21^ and path integral (PI-)AIMD^22^ simulations taking into account the quantum nature of matter have shown the impossibility of electrofreezing liquid water. Nevertheless, under the joint action of pressure (i.e., 5 kPa) and in the presence of fields as intense as 0.23 V/Å, simulations predict the electrofreezing of water toward a novel phase of water ice, known as ice χ.^23^ Although EFs proved their partial order-maker action on the structure of ambient-pressure water,^24^ starting from a strength of ∼0.1–0.3 V/Å the dissociation of water into the oxonium (H_3_O)^+^ and hydroxide (OH)^−^ ions is reported.^21,22,25,26^ These counterions remain stable under the field action in the liquid phase.

Even stronger (∼1–5 V/Å) are the EFs spontaneously present at the proximity of natural oxide surfaces^27,28^ while those produced by dipole fluctuations and ascribed to intermolecular interactions in liquids are on the order of ∼0.1–1 V/Å.^29−32^ Owing to a direct coupling with such local fields, the application of strong external EFs on matter typically produces a range of multifaceted catalytic effects.^33−40^ Despite the contextual presence of ammonia and EFs generated either in planetary atmospheres or on the surfaces of minerals constituting the interior of ice giants and rocky planets, neither experiments nor computations on the effects induced by EFs on liquid ammonia have been reported so far. With the aim of shedding light on this topic of fundamental concern, we have executed a series of AIMD and PI-AIMD simulations of bulk liquid ammonia at *T* = 252 K by sampling different thermodynamic states corresponding to liquid densities in the range [0.65–0.80] g·cm^–3^ under progressively stronger static and homogeneous EFs from the zero-field regime up to 0.70 V/Å.

The main infrared (IR) features of liquid ammonia are associated with the H–N–H bending and with the N–H stretching vibrational modes. As detailed in the Supporting Information (SI), the employed density functional theory (DFT) framework (i.e., BLYP+D3^41−43^) localizes faithfully the center frequencies of the IR-active modes of liquid ammonia in the zero-field regime and with AIMD. Upon exposure of the sample to increasing EFs, several features of the IR spectrum appreciably change. Among them, the most relevant field-induced frequency shifts are exhibited by the symmetric H–N–H bending band (∼1000 cm^–1^) and by the antisymmetric N–H stretching band (∼3400 cm^–1^), as highlighted in Figure 1. While the mean frequency of the symmetric bending mode band is monotonically blue-shifted, the antisymmetric N–H stretching band undergoes a red-shift under the field action. As shown in Figure 1b, in spite of an initial linear shift toward lower frequencies (at a rate of −15 cm^–1^ per 0.1 V/Å), a conspicuous decrement of −65 cm^–1^ of the antisymmetric stretching frequency is recorded between 0.40 and 0.50 V/Å. Such a vibrational Stark effect,^44^ accompanied by more modest field-induced frequency shifts of the symmetric stretching band lying at lower frequencies (∼3300 cm^–1^), leads to a partial coalescence of the two stretching bands toward a single vibrational mode at intense EF regimes, as shown in Figure 1a and Figure S1 of the SI. The application of external EFs leads, hence, to a sizable decrease of the frequencies at which the NH covalent bonds stretch. As a consequence, the NH_3_ H-bond network is significantly strengthened upon field exposure. Besides, starting from a field strength of 0.50 V/Å, the onset of a novel vibrational mode at ∼1900 cm^–1^ is recorded, as displayed by the arrows in Figure 1a and visualized in Figure S1 of the SI. Such a peak is experimentally detected in crystalline phases of ammonia.^45,46^

**Figure 1:**
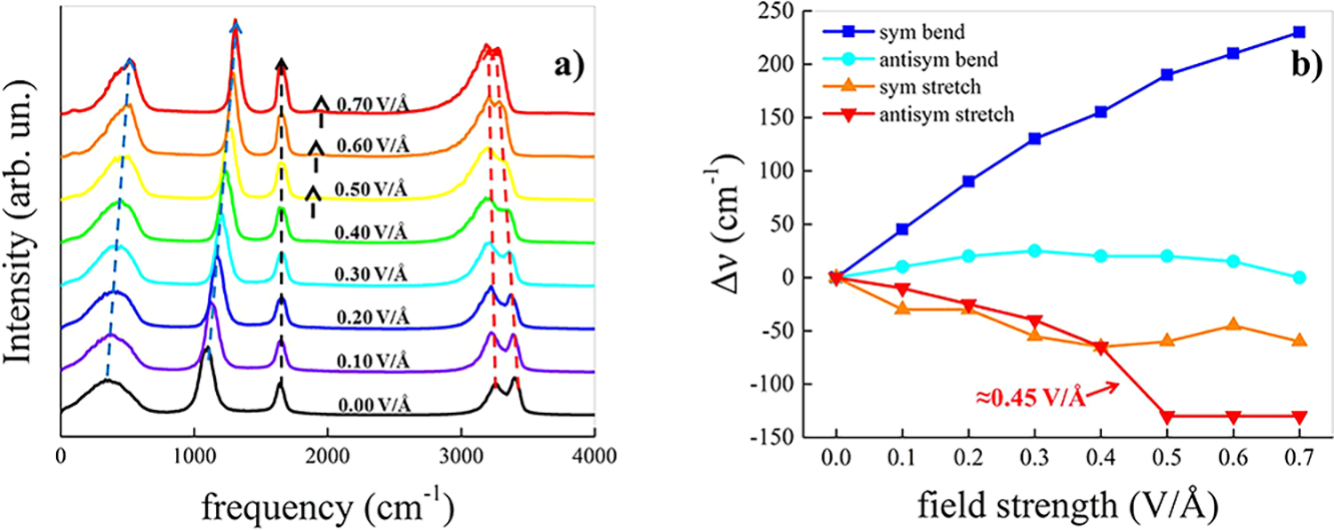
(a) Infrared (IR) spectra of ammonia at *T* = 252 K at zero field (black curve) and for different electric field intensities up to a maximum of 0.70 V/Å. (b) Vibrational Stark effect produced by the application of the field on the bending and stretching modes frequencies.

To track the dynamical response of liquid ammonia to external EFs, the translational self-diffusion coefficient *D* of the ammonia molecules has been calculated. As displayed in the inset of Figure 2, in the absence of the field the self-diffusion coefficient of liquid ammonia at *T* = 252 K determined from our AIMD simulations falls within 2% of the experimental data at the same thermodynamic conditions. In fact, a value equal to 5.83 ± 0.01 × 10^–9^ m^2^·s^–1^ is measured in our simulations *vis-à-vis* an experimental self-diffusion coefficient of 5.95 × 10^–9^ m^2^·s^–1^.^47,48^ It is noteworthy the fact that the inclusion of van der Waals interactions is essential for improving the quality of the first-principles description of the molecular dynamical behavior. As shown in the inset of Figure 2, indeed, values reported in the pioneering work by Boese et al.,^48^ where the BLYP and the HCTH407+ functionals without dispersion corrections were employed, underestimate the self-diffusion coefficient of liquid ammonia by about 9% and 14%, respectively. By switching on the EF, a decrement of the mobility of the ammonia molecules is recorded, as displayed in Figure 2 and in Figure S3 of the SI. In particular, increasing fractions of molecules align their dipole vectors along the field direction. Although the average magnitude of the ammonia dipole moments linearly increases as a function of the field intensity, as shown in Figure S2 of the SI, the self-diffusion coefficient exhibits a 2-fold trend. As reported in Figure 2, a different slope of the curve traced by the self-diffusion coefficient identifies two distinct regimes of molecular mobility depending on the EF strength. In fact, beyond 0.40 V/Å an abrupt collapse of the molecular mobility is recorded, which culminates in a drop of *D* of about 2 orders of magnitude at the maximum field intensity (i.e., from 5.83 × 10^–9^ to 6.83 × 10^–11^ m^2^·s^–1^).

**Figure 2:**
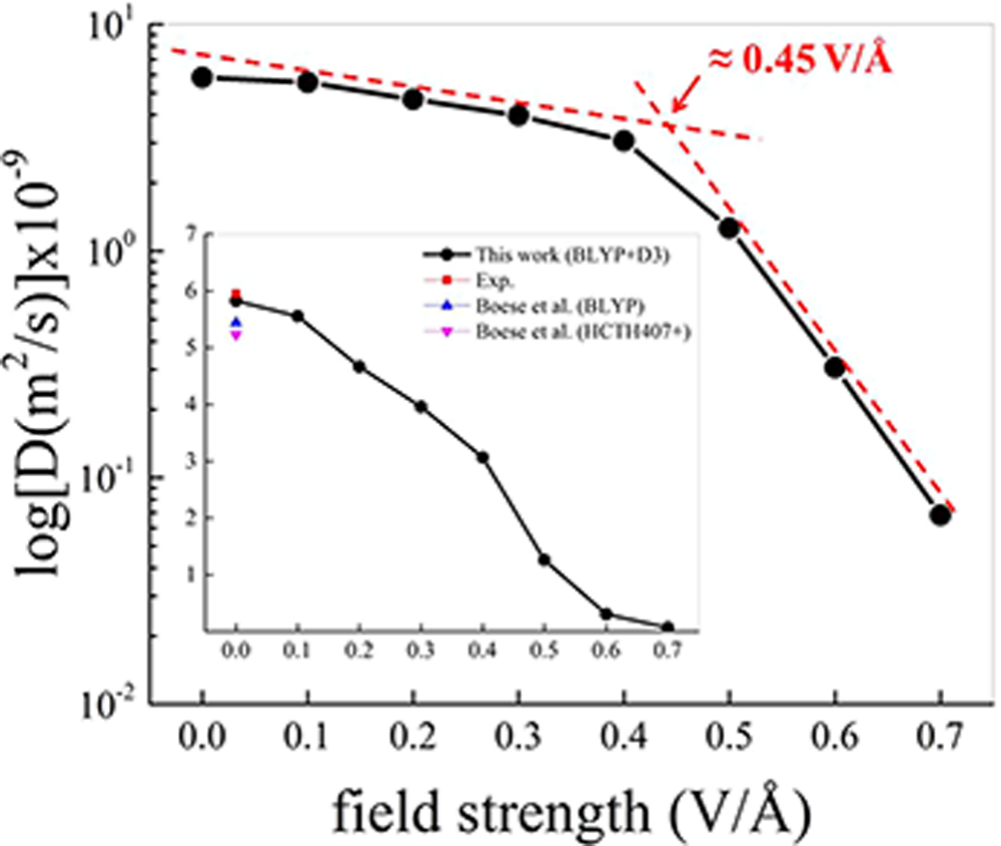
Self-diffusion coefficient of the ammonia molecules as a function of the electric field intensity in logarithmic and (inset) linear scale.

The reduction of the dynamics featuring the behavior of ammonia reflects the field-induced strengthening of the intermolecular interactions. As shown in Figure S4 of the SI, a net modification of the H-bond autocorrelation functions is recorded beyond 0.40 V/Å. The overall H-bond network becomes so persistent that autocorrelation functions assume finite—large—values at long times. Incidentally, the field-induced strengthening of the intermolecular interactions produces spatial correlations between the ammonia molecules that span over sizably longer time scales, as also witnessed by the Van Hove correlation functions *G*(**r**, *t*) (Figure S5 of the SI).

To unveil molecular patterns, the atomistic radial distribution functions (RDFs) for different field strengths have been determined. As displayed in Figure 3 and Figure S6 of the SI, nitrogen–nitrogen (NN) and nitrogen–hydrogen (NH) RDFs are profoundly affected by the application of stronger fields on liquid ammonia. Notwithstanding the overall functional form of the NN RDF in the low-to-moderate field regime remains substantially unaltered (Figure 3), the locations of its peaks and minima are susceptible to the externally applied electrostatic potential gradient. In particular, not only the spatial extent of the first solvation shell of the ammonia molecules is expanded, but also the population of first-neighboring species increases upon the field action, as reported in the inset of Figure 3. In fact, starting from a coordination number of 12.0 in the zero-field case (*vis-à-vis* an experimental value of 12.7^49^), it reaches a maximum value of ∼15.7 for a field strength of 0.40 V/Å, which decreases to ∼5.9 above this field threshold. On the other hand, it has to be noticed that the first local minimum of the NN RDF at strong field regimes exhibits a height larger than 1 and that the integral up to the second (far deeper) minimum leads to a coordination number equal to ∼11.1, as displayed in Figure S8 of the SI. In all cases, the observed discontinuous reentrant effect coincides with the onset of spatially ordered domains identified by the nitrogen atoms of the ammonia molecules in the system. In fact, from a field intensity of 0.50 V/Å, the NN RDFs exhibit a modulated, oscillatory trend (Figure 3): the signature of a field-induced structural transition.

**Figure 3:**
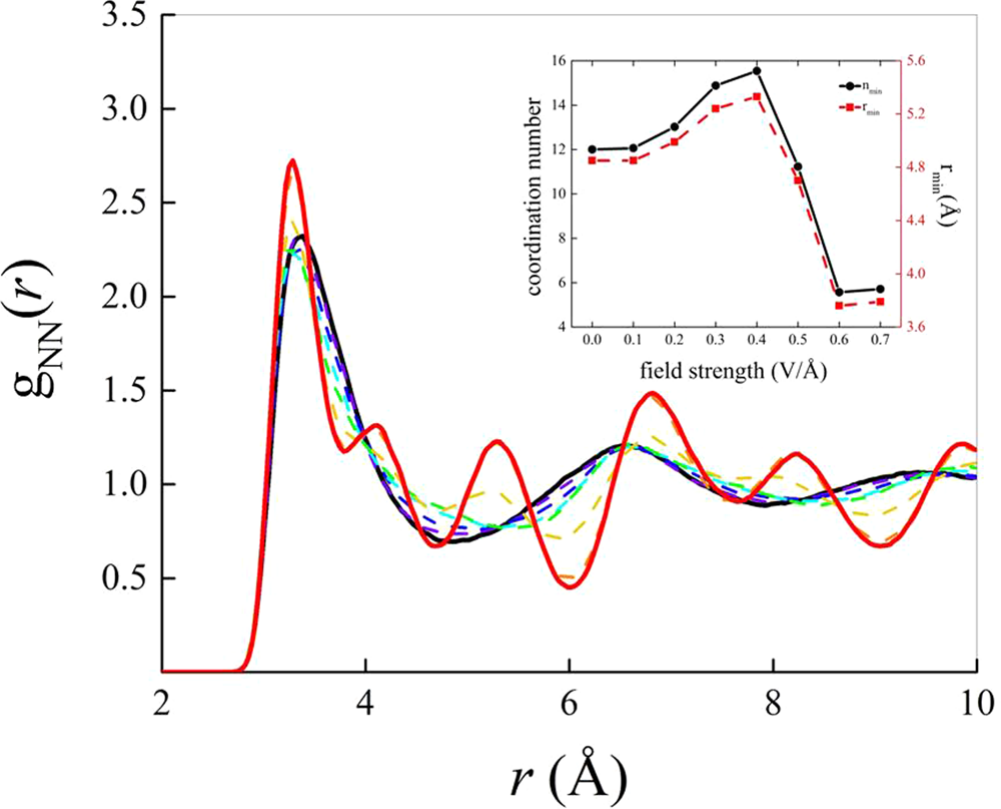
Nitrogen–nitrogen (NN) radial distribution function (RDF) of ammonia for different field intensities from the zero-field regime (black solid curve) up to 0.70 V/Å (red solid curve). Color code follows that of Figure 1a. The inset shows the NN coordination number (black circles) determined as the integral of the RDF up to the first minimum and the location of this latter dip (red squares, red right-handed ordinate axis) as a function of the field strength.

To quantitatively evaluate growing spatial correlations, convenient metrics are given by the translational order parameter *t* defined in ref (50). *t* only modestly depends on the applied field in the low-to-moderate field regime. However, beyond 0.50 V/Å, a previously unexplored region identified by sizably larger values of the translational order parameter and ascribable to the presence of solid-like structures is sampled by the system, as shown in Figure S10b of the SI. Aside from translational order, crystalline structures are featured by orientational order. Among the possible *global* orientational order parameters,^51^ we employed the *q*_6_. In crystalline systems *q*_6_ shows values in the range ∼[0.15–0.65].^52^ Similarly to the translational order parameter, it turns out that in the low-to-moderate field regime also *q*_6_ is somehow insensitive to the application of the external EF. On the other hand, beyond 0.40 V/Å a sudden increase of *q*_6_ is recorded, as shown in Figure S10a of the SI, testifying that orientational correlations between the nitrogen atoms are significantly enhanced by the field. In particular, *q*_6_ exhibits values equal to 0.35 and 0.37 at 0.60 V/Å and 0.70 V/Å, respectively. It follows that these EF regimes are responsible for the freezing of liquid ammonia, in that the original liquid system starts behaving as a structured solid-like sample. By plotting the translational order parameter *t* as a function of the orientational order parameter *q*_6_ for different field strengths, an insightful order map is constructed. As shown in Figure 4, two distinct regions are identified for the condensed phase of NH_3_: a portion—at low-to-moderate field regimes—where the global order is low and ammonia behaves in a fluid-like manner and a region—at intense fields—where the order descriptors assume large values and ammonia shows a solid-like behavior.

**Figure 4:**
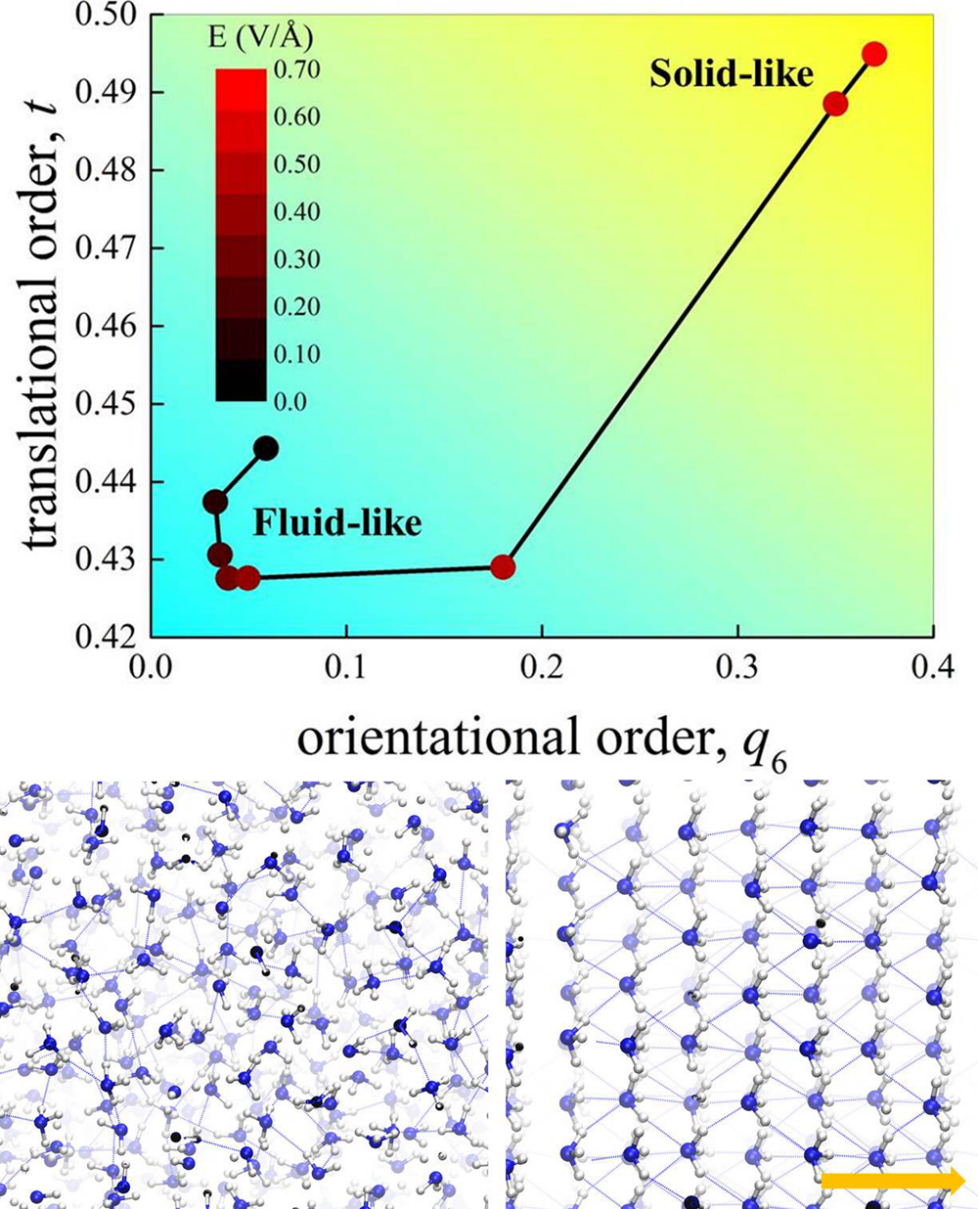
Top panel: order map of ammonia at different electric field intensities (black-to-red gradient scale). Relatively low values of the translational *t* and orientational *q*_6_ order parameters identify a fluid-like state of ammonia (a liquid), whereas large values of *t* and *q*_6_ are the manifestation of a field-induced solid-like ammonia structure. Bottom panels: snapshots of the simulation box in the absence (left) and in the presence (right) of the field producing the stacking of the molecules in layers approximately perpendicular to the field direction, the latter being represented by the yellow arrow. In spite of the ferroelectric nature conferred by the field (i.e., all NH_3_ molecules are oriented along the field axis), hydrogen atoms remain disordered.

In net contrast with the *chemistry* activated by fields on the order of ∼0.1–0.3 V/Å in water,^21,22,25,26^ it turns out that intense EFs up to 0.7 V/Å are not capable of dissociating ammonia molecules. Conversely, the EF drives a *physical* modification of the whole H-bond structure toward a ferroelectric solid-like system, where the nitrogen atoms occupy the positions of a regular lattice while molecular dipoles are predominantly aligned toward the field axis, as shown in the bottom panels of Figure 4. Since Nuclear Quantum Effects may play a role in lowering the free-energy barrier for molecular dissociation and proton transfer^22^—especially in a system composed of a large number of (light) hydrogen atoms and at a relatively low temperature—we have included the zero-point motion and tunnelling effects of the nuclei in a series of PI-AIMD simulations. As shown in Figure S7 of the SI, the electrofreezing of the liquid takes place even when, additionally to electrons, also nuclei are treated as quantum entities.

As detailed when commenting on Figure 3, the number of first neighbors decreases to ∼5.9 at the highest field regime. Such a coordination number is typical of simple cubic structures. In addition, a value of *q*_6_ ∼ 0.35 observed after the structural transition corroborates the hypothesis of a cubic symmetry.^52^ However, the structure found is different from that typical of phase I not only by virtue of the ferroelectric orientation of the molecular dipoles but also because other domains exhibiting an apparent pseudo-*fcc* packing and distorted (chairlike) hexagonal symmetry are recorded, as shown in Figure S13 and Figure S14 of the SI, respectively. In fact, the coordination number determined up to the second minimum of the NN RDF at intense fields—much more pronounced than the first one—is ∼11.1 (Figure S8 of the SI), a value compatible with the *ABCABC* stacking (Figure S13) of the nitrogen sublattice.

A plausible rationale behind the differently observed response to static EFs of ammonia with respect to other H-bonded systems may be found in the peculiarities of its H-bond network. Each NH_3_ molecule in the liquid tends to donate and accept on average ∼1.3 H-bonds.^48^ Considering that in the first solvation shell about 12 ammonia molecules are present, it is clear that steric effects play a more major role than (quite feeble) H-bonds. Moreover, the fact that each ammonia molecule could in principle be capable of donating 3 protons while presenting only 1.3 H-bonds sizably reduces the possibility for proton transfer. In fact, other investigated H-bonded liquids, which dissociate and do not freeze under the field action, all shows an equal number of potentially available protons and lone pairs. Water, indeed, presents 2 lone pairs and holds 2 protons, one of which can be transmitted—upon field exposure—through one of the ∼2 H-bonds each H_2_O molecule donates in the liquid.^21,22^ Similarly, dissociable simple alcohols, on their hydrophilic heads, hold 1 proton donating ∼1 H-bond.^53,54^ This way, while other H-bonded systems dissipate the energetic contribution carried by EFs by maximizing the entropy via concerted proton transfers owing to a tailored coupling between the number of charge carriers (H^+^) and transmitting pathways (H-bonds/lone pairs), proton-rich but poorly H-bonded liquid ammonia finds a novel nitrogen-ordered ferroelectric configuration under the field action.

This study proves that, like extreme temperature and pressure regimes, intense EFs can be used as a means for reaching previously unexplored regions in phase diagrams. This way, our findings indicate that the phase diagram of ammonia under planetary conditions may be far richer than previously expected. Finally, the possibility of realizing unprecedently observed ferroelectric solid phases of ammonia paves the way toward the development of safe storage techniques and transportation strategies for, e.g., hydrogen production.

## Supporting Information Available

Computational details; IR spectra; molecular dipole moments distributions; mean square displacements; H-bond autocorrelation functions; Van Hove correlation functions; additional radial distribution functions; additional spatial correlations; order parameters as a function of the field strength; asphericity of the Voronoi cell; and snapshots from *ab initio* molecular dynamics trajectories (PDF)
