# Supplementary material for: Electrofreezing of Liquid Ammonia
Source: J Phys Chem Lett. 2022 Oct 18;13(42):9889–94. doi: 10.1021/acs.jpclett.2c02576 (PMC9619927; doi:10.1021/acs.jpclett.2c02576)
Supplement: Supplementary file 1 — jz2c02576_si_001.pdf [file jz2c02576_si_001.pdf]

Supporting Information
Electrofreezing of Liquid Ammonia

Giuseppe Cassone,[1*] Jiri Sponer[2,3], Judit E. Sponer[2], Franz Saija[1]

[1]Institute for Chemical-Physical Processes, National Research Council of Italy, Viale F. Stagno d'Alcontres 37, 98158 Messina (Italy)

[2]Institute of Biophysics of the Czech Academy of Sciences, Královopolská 135, 61265 Brno (Czechia)

[3]Regional Center of Advanced Technologies and Materials, The Czech Advanced Technology and Research Institute (CATRIN), Palacky University Olomouc, Slechtitelu 27, 77900 Olomouc (Czechia)

E-mail: cassone@ipcf.cnr.it

(Dated: October 13, 2022)

I. COMPUTATIONAL DETAILS

A. *Ab initio* molecular dynamics

The software package CP2K [1], based on the Born-Oppenheimer approach, was used to perform *ab initio* molecular dynamics (AIMD) and path integral AIMD (PI-AIMD) [2] simulations of bulk liquid ammonia within a constant number, volume, and temperature (NVT) canonical ensemble. Samples of liquid ammonia under the action of external static and homogeneous electric fields oriented along the z-axis were simulated both *via* classical nuclei AIMD and PI-AIMD including Nuclear Quantum Effects (NQEs).

Liquid ammonia samples composed of 64 (256 atoms), 128 (512 atoms), and 256 (1024 atoms) NH_3 molecules were simulated each at three different densities (*i.e.*, 0.65 g·cm^{-3}, 0.73 g·cm^{-3}, and 0.80 g·cm^{-3}). The systems composed of 64 molecules were arranged in cubic cells with edges equal to 14.07 Å, 13.54 Å, and 13.13 Å reproducing densities of 0.65 g·cm^{-3}, 0.73 g·cm^{-3}, and 0.80 g·cm^{-3}, respectively. Simulation samples composed of 128 molecules were arranged in cubic cells with edges equal to 17.73 Å, 17.05 Å, and 16.54 Å reproducing densities of 0.65 g·cm^{-3}, 0.73 g·cm^{-3}, and 0.80 g·cm^{-3}, respectively. Simulation boxes composed of 256 molecules were arranged in cubic cells with edges equal to 22.33 Å, 21.48 Å, and 20.84 Å reproducing densities of 0.65 g·cm^{-3}, 0.73 g·cm^{-3}, and 0.80 g·cm^{-3}, respectively. In addition, tests with a sample composed of an odd number of molecules (*i.e.*, 101 (404 atoms)) were performed at a density of 0.80 g·cm^{-3}. The results reported in the main text refer to the simulation box containing 256 molecules at a density of 0.73 g·cm^{-3}.

As usual, in order to minimize spurious and nonphysical surface effects, the structures were replicated in space by employing periodic boundary conditions. Intensities of the electric fields were gradually increased with a step increment of 0.05 V/Å from zero up to a maximum of 0.70 V/Å. Whilst dynamics of 20 ps were performed in all zero-field cases, for each other value of the field intensity dynamics of 20 ps were ran for the simulated samples up to 128 molecules. For the biggest samples containing 256 NH_3 molecules, trajectories of 10 ps for each value of the field strength were accumulated. Tests aimed at checking the convergence of the presented results with longer simulations at a given field intensity (*i.e.*, 100 ps) were executed. Besides, additional long simulations ($\geq$ 100 ps) were ran to check the hysteresis upon switching off of the field. In all cases, solid-like structures returned into

the liquid state within tenths of ps.

Wavefunctions of the atomic species have been expanded in TZVP basis sets with Goedecker-Teter-Hutter pseudopotentials using the GPW method [3]. A plane-wave cutoff of 400 Ry has been imposed. Exchange and correlation (XC) effects were treated with the gradient-corrected Becke-Lee-Yang-Parr (BLYP) [4] density functional. In order to take into account dispersion interactions, the dispersion-corrected version of BLYP (*i.e.*, BLYP+D3(BJ)) [5] was employed. Simulations were carried out at the nominal temperature of 252 K, similarly to other computational investigations [6]. In the AIMD simulations, the dynamics of the nuclei was simulated classically using the Verlet algorithm whereas the canonical sampling has been executed by employing a canonical-sampling-through-velocity-rescaling thermostat [7] set with a time constant equal to 20 fs.

B. Path integral *ab initio* molecular dynamics

Path integral *ab initio* molecular dynamics (PI-AIMD) [2] simulations with a colored noise thermostat [8] of bulk liquid ammonia within a constant number, volume, and temperature (NVT) canonical ensemble were also performed with the aim of evaluating the role carried by Nuclear Quantum Effects (NQEs) in eventually preventing the electrofreezing observed in the classical nuclei AIMD simulations. NQEs may in principle play an important role in lowering the free-energy barrier for molecular dissociation and proton transfer [9]. The PI-AIMD sampling was obtained in the framework of the generalized Langevin equation thermostat (PIGLET) method [8] using six beads per atom. Such a thermostating technique, by accelerating the convergence, reduces the number of beads needed for mapping the classical system onto the quantum one [10]. All results refer to the centroid of the ring polymer beads. Being computationally more demanding than AIMD simulations, only the systems composed of 64 NH_3 molecules (*i.e.*, 256 atoms) were simulated at the different densities reported in the previous section. Trajectories of 10 ps for each field intensity were gathered whereas 20-ps-long trajectories were accumulated in the zero-field regimes leading to a cumulative simulation time equal to $\sim$ 500 ps. An imaginary time-step of 0.5 fs for each bead of the PI-AIMD simulations was employed. Moreover, in the zero-field case, also tests employing imaginary time-steps equal to 0.4 fs and 0.3 fs were carried out.

C. Electric field implementation

The implementation of an external field in computations based on Density Functional Theory (DFT) can be achieved by exploiting the Modern Theory of Polarization and Berry's phases [11]. In a nutshell, the electrostatic interactions are determined by first-principles, as reported by Umari and Pasquarello [12]. For instance, they demonstrated that the functional $F = E_{KS}(\{\psi_{kn}\}) - \epsilon \cdot \mathbf{P}(\{\psi_{kn}\})$, where E_{KS} is the Kohn-Sham energy functional, ϵ is the field intensity, and $\mathbf{P}$ is the polarization, is exploitable as energy functional for a variational approach to the finite-field problem as well.

II. ADDITIONAL RESULTS

Infrared (IR) spectra shown in Fig. 1 of the main text and Fig. S1 here have been determined by means of the software TRAVIS [13, 14] from the centres of the Maximally Localized Wannier Functions (MLWFs) [15, 16] calculated on the fly during the *ab initio* molecular dynamics (AIMD) simulations. Molecular dipoles from MLWFs centers can be determined as:

$$\mu = -2e \sum_i \mathbf{r}_i + e \sum_j Z_j \mathbf{R}_j \,, \tag{1}$$

where e is the electron charge, $\mathbf{r}_i$ is the position vector of the MLWF center i, Z_j is the atomic number of the nuclei j whilst $\mathbf{R}_j$ is the position vector of this latter. This way, the IR spectra at all the investigated field intensities of liquid ammonia were computed as the Fourier transform of the molecular dipole autocorrelation function along the simulation trajectories.

Experiments locate the IR vibrational bands ascribed to the symmetric and antisymmetric bending modes at 1050 and 1630 cm^{-1}, respectively [17]. As shown in Fig. 1 of the main text (black curve) and in Fig. S1 here, the employed Density Functional Theory (DFT) framework (*i.e.*, BLYP+D3 [4, 5]) localizes faithfully the center frequencies of the bending modes of liquid ammonia in the zero-field regime. In fact, whereas the frequency peak of the symmetric bending mode falls at 1080 cm^{-1}, the antisymmetric one is located around 1640 cm^{-1}, giving rise to a maximum error smaller than 3% with respect to the experimental data. Furthermore, the excellent agreement of our computations at zero-field regime with laboratory experiments results is also witnessed by the comparison of the location of

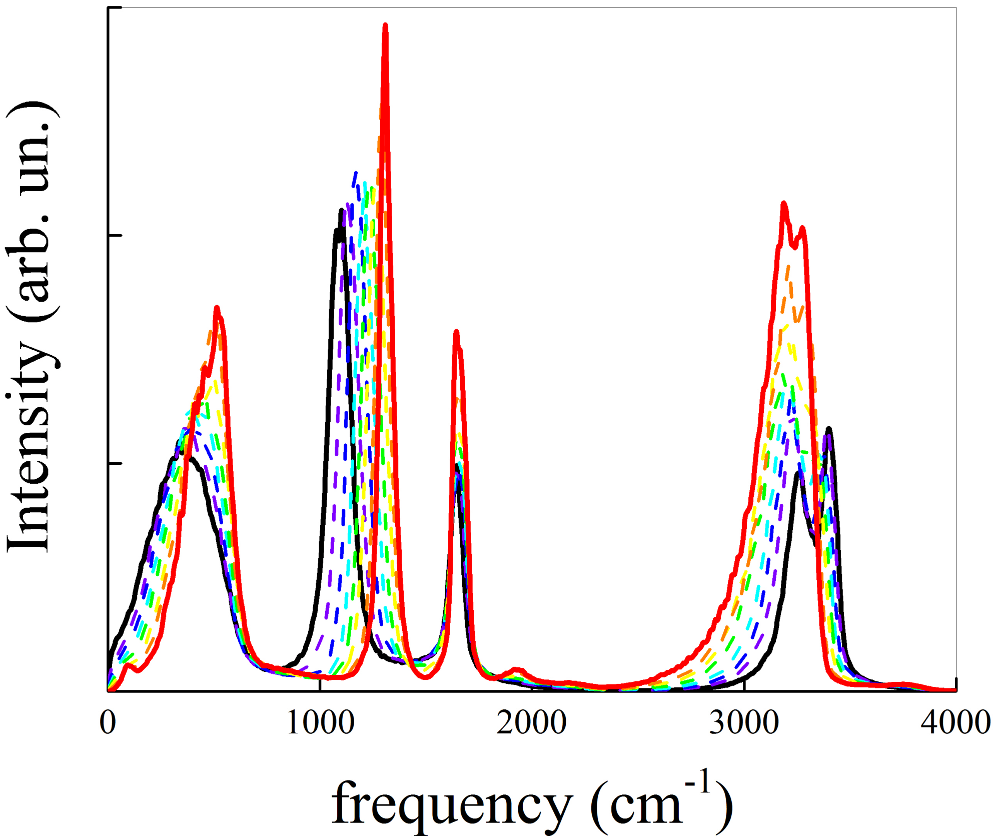

FIG. S1: Infrared (IR) spectrum of ammonia at $T = 252$ K at zero field (black solid curve) and for different electric field intensities up to 0.70 V/Å (red solid curve). Dashed curves refer to 0.10 V/Å (purple), 0.20 V/Å (blue), 0.30 V/Å (cyan), 0.40 V/Å (green), 0.50 V/Å (yellow), and 0.60 V/Å (orange) cases, in accordance with the IR spectra presented in Fig. 1 of the main text.

the frequency peaks ascribed to the antisymmetric and symmetric N-H stretching bands. In fact, whilst IR experiments of liquid ammonia locate the antisymmetric and symmetric stretching at 3380 and 3240 cm^{-1} [17], respectively, our first-principles simulations find peak frequencies equal to 3395 and 3255 cm^{-1} in this spectral region. This finding indicates that the treatment of the molecular dipoles correlations and the overall simulation setup (*i.e.*, box size, trajectory length, etc.) – producing a discrepancy lower than $\sim 1\%$ when compared to experiments – adequately describe subtle intra and intermolecular interactions in liquid ammonia. Thus, although neglecting Nuclear Quantum Effects (NQEs) in classical nuclei

AIMD simulations generally results in blue-shifted stretching vibrations [18], also by virtue of errors compensation effects the BLYP+D3 functional appears to work particularly well for liquid ammonia.

As visible from the IR spectra of Fig. 1 of the main text and Fig. S1 here, the progressive growth of a high-frequency side mode of the low-frequency band is the manifestation of the additional topological constraint imposed by the field on the molecular dipoles. This vibrational Stark effect has recently been investigated in low-temperature (*i.e.*, 10 K) ammonia molecules freely rotating in a Ar matrix and exposed to a DC electric field [19]. This way, ammonia molecules start librating rather than rotating under sufficiently intense electric fields. Besides, a contraction of the entire frequency range in the IR spectrum is recorded upon raising the field, an aspect appearing to characterize the general vibrational response of H-bonded liquids to externally applied electric fields. In other words, whereas the high-frequency bands are shifted by the field at lower frequencies, the low-frequency bands are blue-shifted, as shown in Fig. 1 of the main text and in Fig. S1.

Owing to the coupling between the external electrostatic potential gradient and the local molecular dipoles, another crucial outcome emerges when static fields are applied on H-bonded systems. In fact, as visible from Fig. S4, the dynamics of the H-bond network is slowed down, an aspect reflecting the field-induced strengthening of the intermolecular interactions. A rough estimate of the energetic enhancement of the H-bonds can be extracted from the field-induced red-shift of the IR NH stretching mode band. Accordingly to investigations on the water H-bond dynamics and to second-generation Car-Parrinello molecular dynamics simulations of water [20], the field-induced NH stretching frequency decrease of ~ 130 cm^{-1} recorded at the maximum field intensities here investigated can be ascribed to an average increase of $\sim 7-8$ kJ/mol of the H-bond strength. This means that the electric field – in conjunction with the induced dipoles reorientation, consequent dipole-dipole coupling and local polarization effects – is capable of reinforcing each H-bond of about 60%, a value close to that recorded in bulk liquid water under static electric fields [21].

To track, at the molecular scale, the dynamical response of liquid ammonia to external static and homogeneous electric fields, the translational self-diffusion coefficient D of the ammonia molecules has been calculated from the mean square displacement (MSD) as

$$D = \lim_{t\to\infty} \frac{\langle [\mathbf{r}(t) - \mathbf{r}(0)]^2 \rangle}{6t}\,, \tag{2}$$

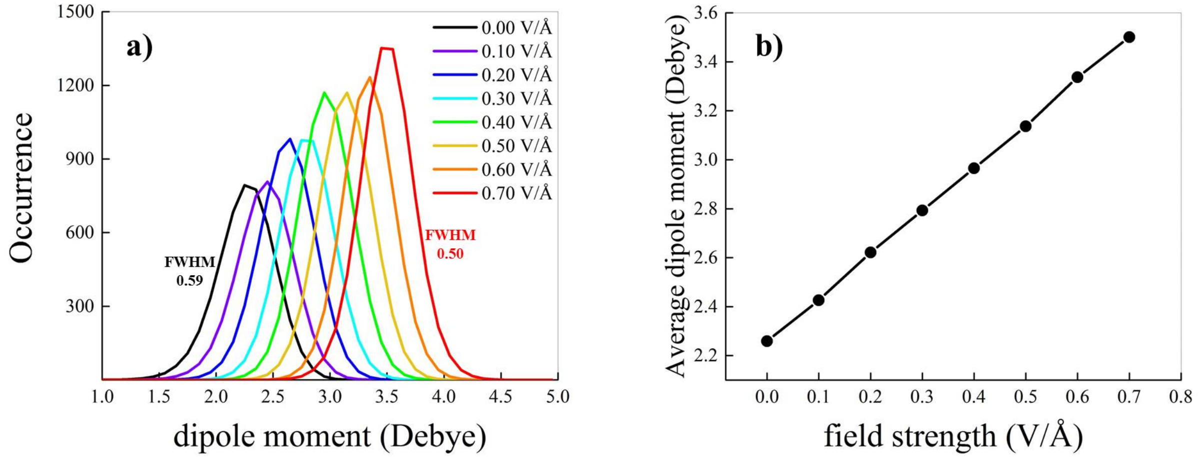

FIG. S2: (a) Distribution of the molecular dipole moments in ammonia at $T = 252$ K in the zero-field regime and for different field intensities (see legend). Full widths at half maximum (FWHM) of the extreme cases highlight the field-induced narrowing of the distributions. (b) Average molecular dipole moment of ammonia as a function of the field intensity.

where $\mathbf{r}(t)$ is the position of the center of mass of a molecule at time t. In particular, a linear regression has been performed after excluding the initial ballistic behavior of the MSD within the first ~ 0.5 ps.

Similarly to other works dealing with ammonia [6] and other H-bonded liquids [22], a geometric criterion has been adopted to identify H-bonded molecules in the simulated systems. We performed a structural analysis of the H-bond network and identified an H-bond through the following geometric conditions (that must be simultaneously fulfilled): two ammonia molecules are considered as H-bonded if $R^{(NN)} < R_c^{(NN)}$, $R^{(NH)} < R_c^{(NH)}$, and $\angle$N-H$\cdots$N$< 30°$, where R is the instantaneous distance between the respective atoms and R_c is the distance cutoff determined from the positions of the minimum of the respective intermolecular RDFs. We calculated also the time autocorrelation function of H-bonds as:

$$C_{HB}(t) = \frac{\sum_{\langle i,j \rangle} s_{ij}(t_0) s_{ij}(t_0 + t)}{\sum_{\langle i,j \rangle} s_{ij}(t_0)}\,, \tag{3}$$

where the indices i and j run on all pairs of first-neighbor molecules which at t_0 were H-bonded, t_0 being the time at which the measurement process begins; $s_{ij} = 1$ if the criterion for the presence of an H-bond is fulfilled, $s_{ij} = 0$ otherwise. The results were averaged over thousands initial configurations.

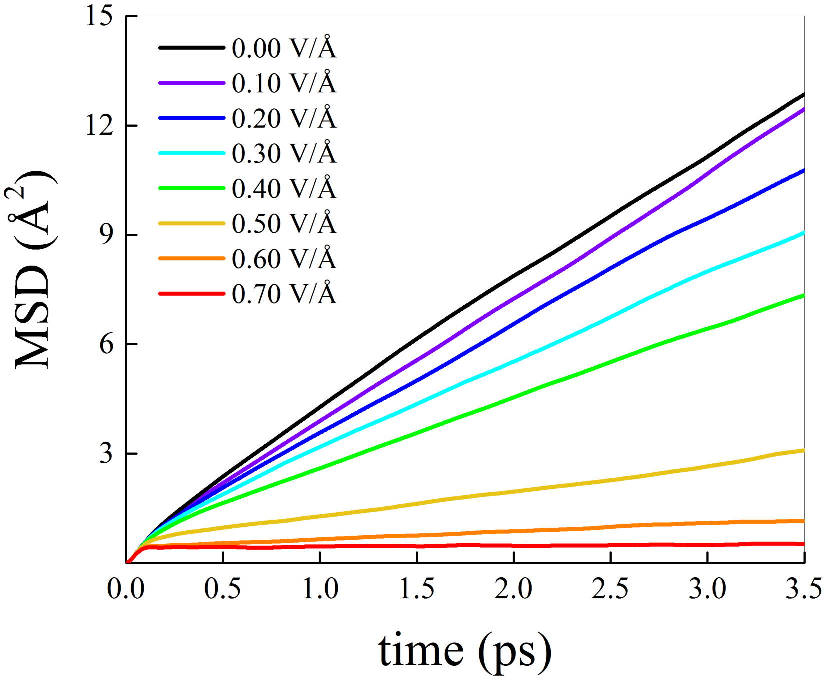

FIG. S3: Mean square displacement (MSD) of the nitrogen atoms of the ammonia molecules in the zero-field case and for several field strengths (see legend).

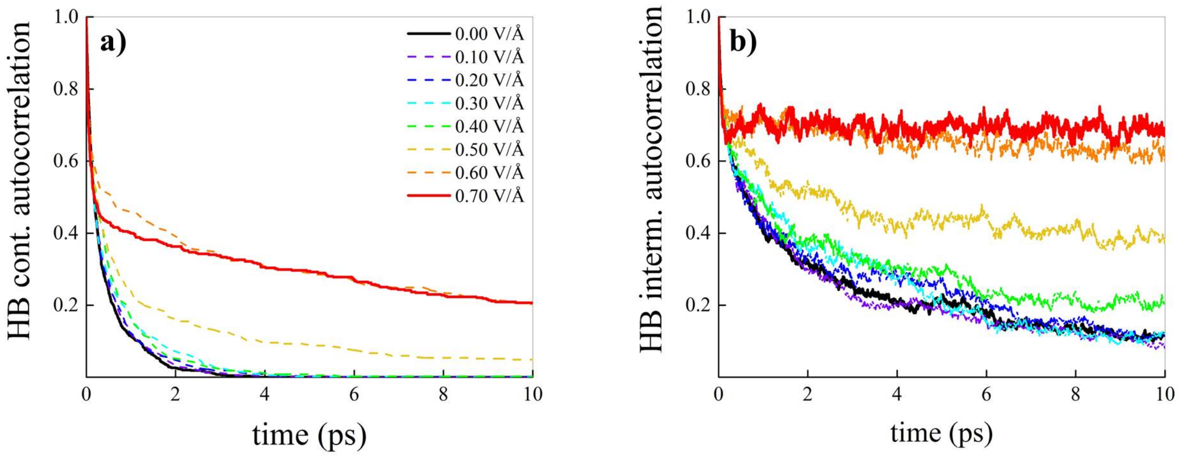

FIG. S4: (a) Continuous autocorrelation function of H-bonds at zero field and at different field intensities (see legend). (b) Intermittent H-bond autocorrelation function determined by allowing a given H-bond to cleave within timescales ≤ 5 fs and assigning to s_{ij} a value equal to 1 (see eq. (3)).

It turns out that the (continuous) autocorrelation function of the H-bonds is only

marginally affected by the electric field rise up to a strength of 0.40 V/Å. As shown in Fig. S4-a, a net modification of the shape of the H-bonds continuous autocorrelation function is recorded beyond this latter threshold. In fact, the continuous H-bond lifetime doubles from $\sim$ 250 fs beneath 0.40 V/Å to $\sim$ 500 fs at 0.50 V/Å. More importantly, the overall H-bond network becomes so persistent that, above this latter intensity, a lifetime of $\sim$ 2 ps is observed along with the evidence that autocorrelation functions assume large finite values at longer times, as visualized in Fig. S4-b also for the more tolerant intermittent H-bond autocorrelation function.

Van Hove correlation functions $G(\mathbf{r}, t)$ are particularly useful because their two-dimensional Fourier transform corresponds to the dynamic structure factor $S(\mathbf{q}, \omega)$, which can be measured by neutron scattering experiments. To monitor molecular correlations we have calculated the temporal decay of the $G^1_{NN}(t)$, a quantity measuring the correlations between first-neighbors nitrogen atoms over time (Fig. S5-c). Considering that when no correlations are present $G^1_{NN}(t) \sim 1$, it follows that in the zero-field regime structural correlations between first-neighboring ammonia molecules vanish more rapidly than in presence of a finite field, as shown in Fig. S5-c. In addition, the field-induced enhancement of the spatial and temporal correlations is not merely referred to the nearest-neighbor species but applies at all scales, as visible from a direct comparison of the Van Hove correlation function between nitrogen atoms $G_{NN}(\mathbf{r}, t)$ at zero field (Fig. S5-a) and upon applying a field intensity of 0.50 V/Å (Fig. S5-b). Typical intermolecular correlations occurring at different distances persist, indeed, over the simulated timescales in presence of the field, as visible from the red/yellow/orange regions in the contour map of the Van Hove correlation function plotted in Fig. S5-b. By contrast, a diffuse green area associated with $G_{NN}(\mathbf{r}, t) \sim 1$ testifies the rapid temporal decay of the spatial structural correlations in liquid ammonia in absence of the field due to the presence of weak H-bonds (Fig. S5-a).

The Voronoi cell of individual ammonia molecules was widely used to characterize local structures in liquid systems. Asphericity of the Voronoi cell parameter η features the shape of the resulting Voronoi cell and is given by the following expression:

$$\eta = \frac{A^3}{36\pi V^2}\,, \tag{4}$$

where V is the Voronoi cell volume and A is its surface area. Asphericity is here calculated based on the Voronoi analysis on the nitrogen atoms of ammonia. This unit-less parameter is

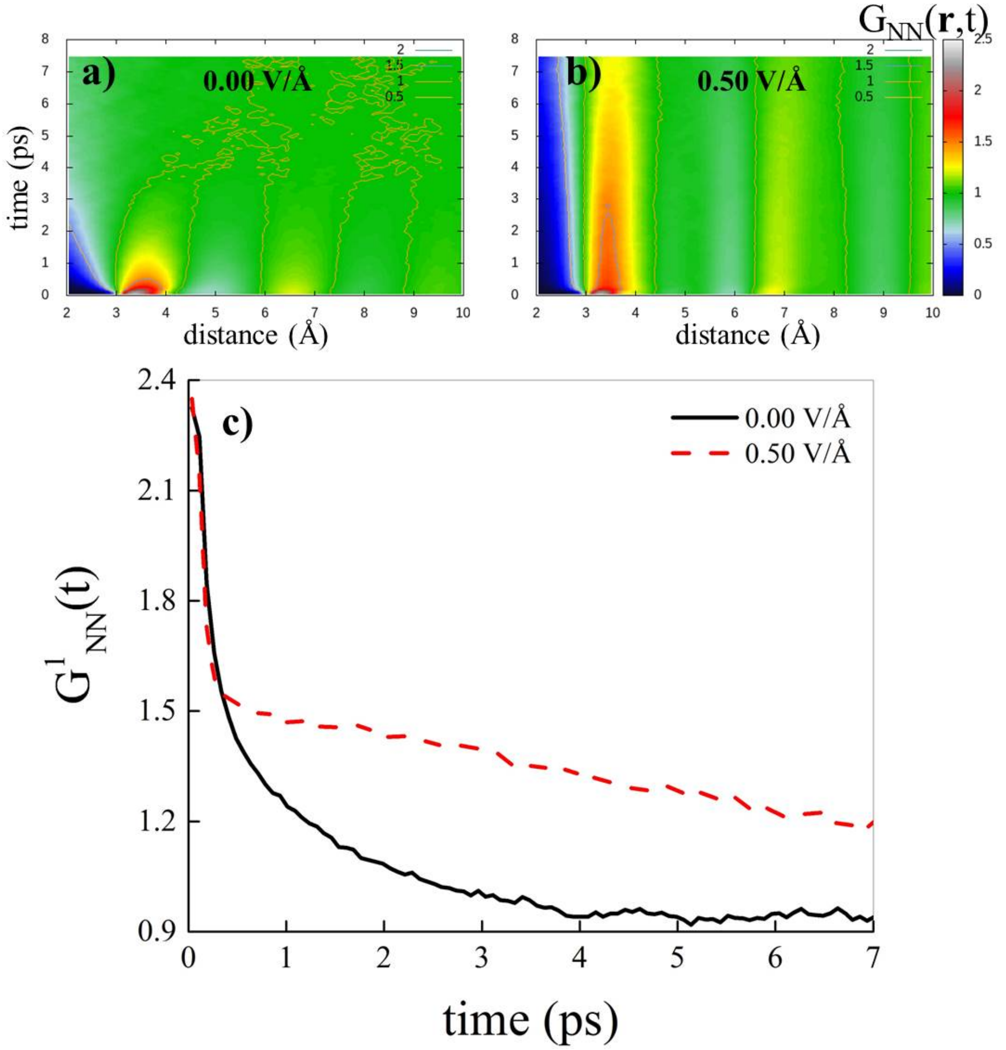

FIG. S5: (a) and (b) display the partial Van Hove correlation function between the nitrogen atoms (*i.e.*, $G_{NN}(\mathbf{r}, t)$) as a function of the time and of the intermolecular distance in absence of the field and in presence of a static electric field with an intensity of 0.50 V/Å, respectively. (c) Temporal dependence of the Van Hove $G^1_{NN}(t)$ function of the nitrogen atoms at zero field (black curve) and at 0.50 V/Å (red dashed curve).

1 for a perfect sphere. The parameter η characterizes the shape of the Voronoi cell in a size-independent manner. For the sake of completeness, we have determined the distribution of the apshericity of the Voronoi cell as a function of the external electric field intensity, proving that the field acts as an order-maker agent in the ammonia structure (see Fig. S11). On the other hand, it is worth remarking that the orientational order parameter q_6 is far more sensitive to the field-induced structural changes than the asphericity of the Voronoi

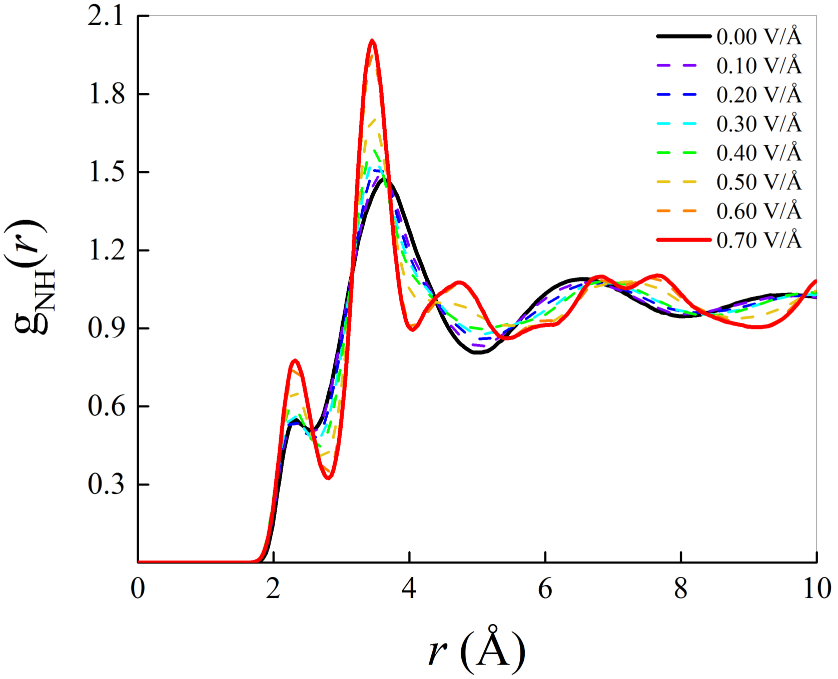

FIG. S6: Nitrogen-hydrogen (NH) radial distribution function of ammonia at $T = 252$ K and for different field strengths in the zero-field regime and for different field strengths (see legend).

cell η (see Fig. S10-a).

To quantitatively evaluate growing spatial correlations in the simulated systems we hence employed the translational order parameter t defined as [23]:

$$t = \frac{\int_0^{\xi_c} |h(\xi)| d\xi}{\xi_c} \quad . \tag{5}$$

In this expression density-density correlations are detected by integrating over the absolute value of the total correlation function $h(\xi) = g(\xi) - 1$, where $g(\xi)$ is the RDF determined as a function of the rescaled spatial coordinate $\xi = r\rho^{1/3}$ and ξ_c is a numerical cutoff imposed by the finite size of the simulation box (*i.e.*, $\xi_c \sim 3$ in our case). The strength of this choice resides in the fact that it represents a measure of the order which is independent from the specific crystalline symmetry, this latter being *a priori* unknown. Fig. S10-b shows the trend of t as a function of the field strength.

Aside from translational order, crystalline structures are featured by orientational order.

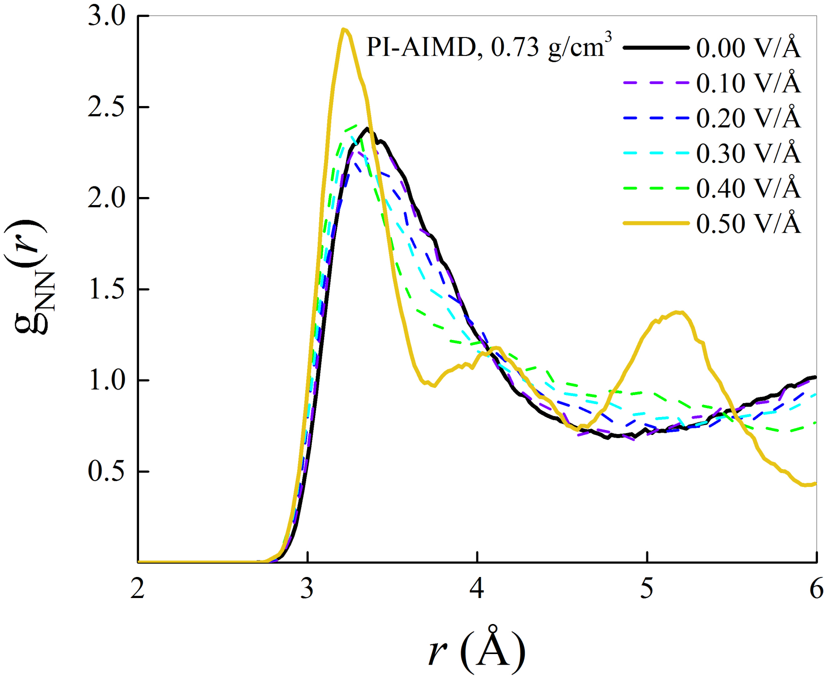

FIG. S7: Nitrogen-nitrogen (NN) radial distribution function of ammonia at $T = 252$ K and for different field strengths from the zero-field regime and for different field strengths (see legend), as determined from path integral *ab initio* molecular dynamics simulations (PI-AIMD) taking into account Nuclear Quantum Effects (NQEs).

Let us consider the *global* orientational order parameter defined as [24]:

$$q_l = \left(\frac{4\pi}{2l+1} \sum_{m=-l}^{m=l} |\overline{Q}_{lm}|^2 \right)^{1/2} , \tag{6}$$

where $\overline{Q}_{lm} = \langle Y_{l,m}(\theta_{i,j}, \phi_{i,j}) \rangle$ is the average of the spherical harmonics $Y_{l,m}$ of *all* bonds connecting the nitrogen atoms in the system. q_6 is the orientational order parameter obtained by imposing $l = 6$ and it was chosen in the current investigation to monitor the field-induced increase of the orientation order in liquid ammonia (see Fig. S10-a).

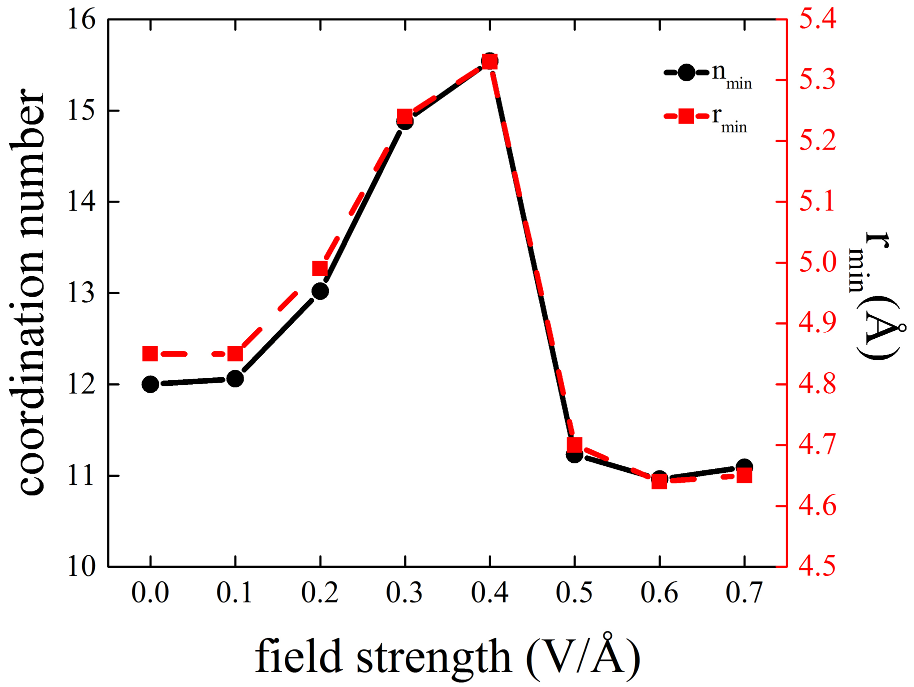

FIG. S8: Nitrogen-nitrogen coordination number (black circles) determined as the integral of the respective radial distribution function (see Fig. 3 of the main text) up to the first significant minimum having an height lower than 1, and location of this latter dip (red squares, red right-handed ordinate axis) as a function of the field strength.

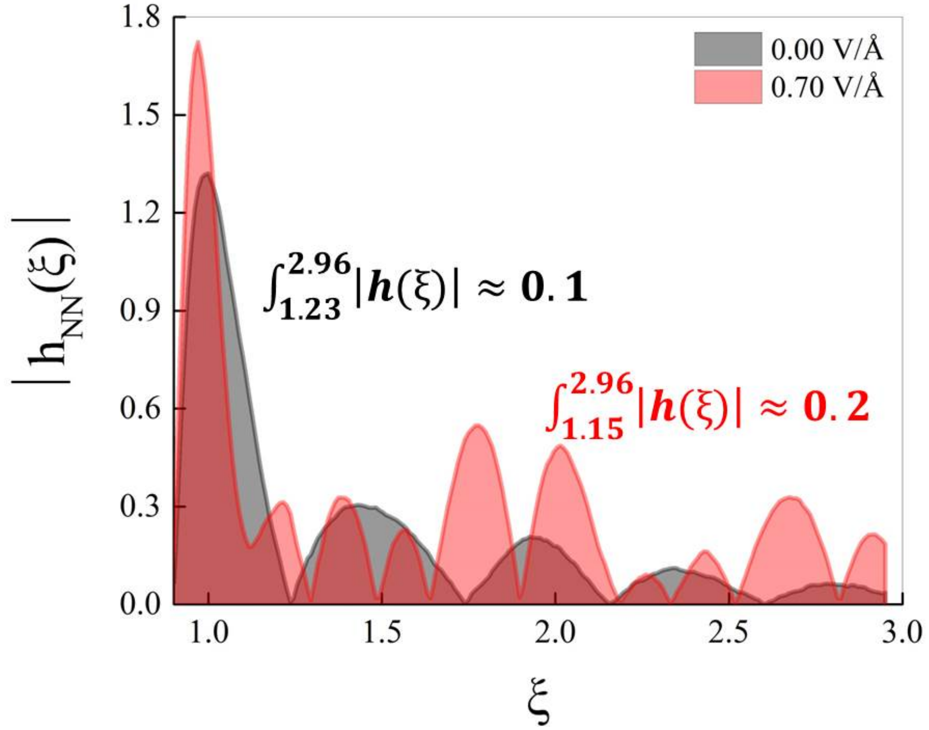

FIG. S9: Absolute value of the total correlation function between the nitrogen atoms of ammonia $h_{NN}(\xi)$ determined in the zero-field regime (grey curve) and for a field intensity of 0.70 V/Å (red curve). Since integrals of this function are directly related to the translational order in the system, the values of the respective integrals along with the corresponding areas are displayed.

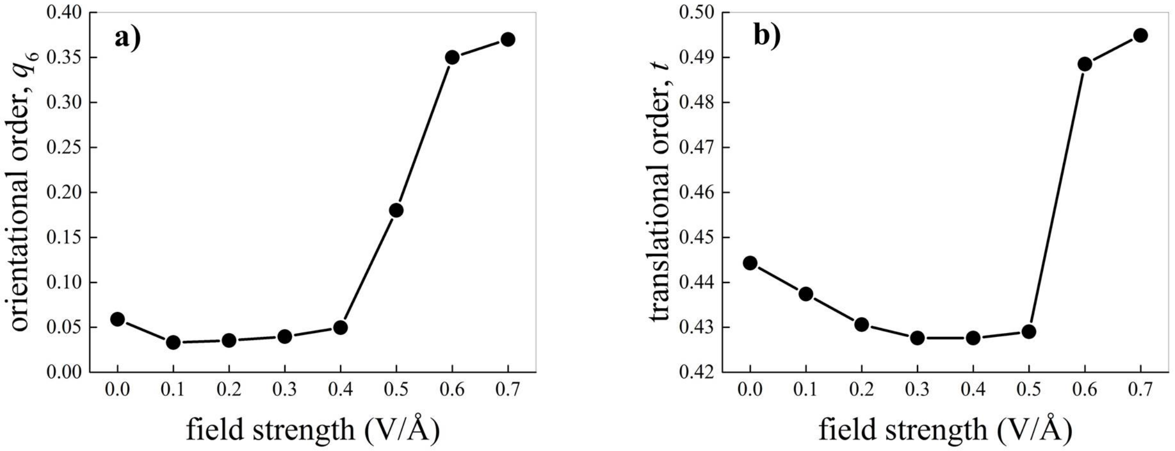

FIG. S10: (a) Orientational order parameter q_6 and (b) translational order parameter t plotted as a function of the field strength.

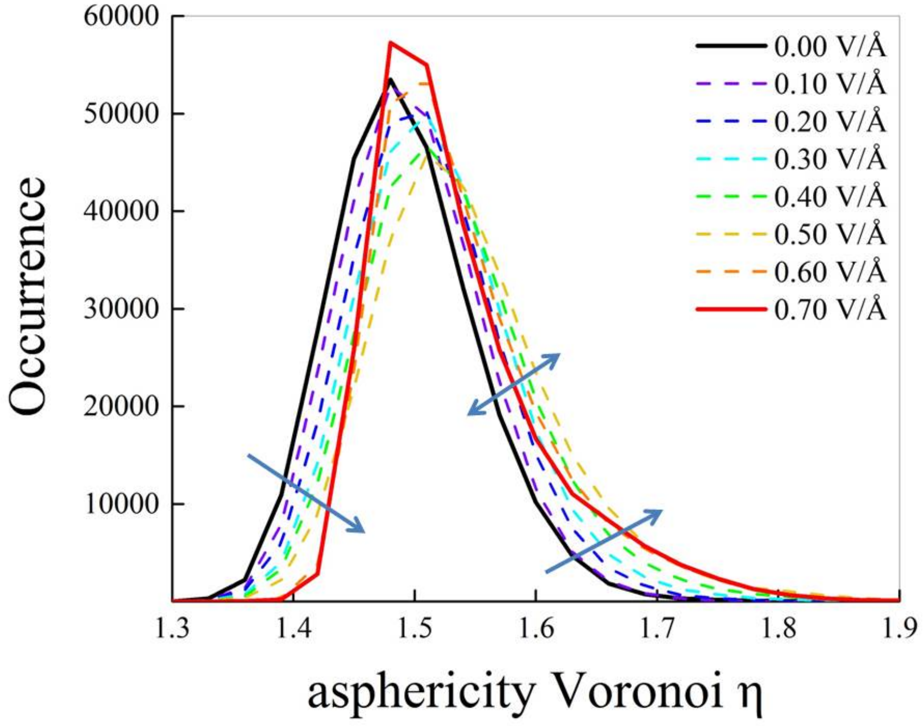

FIG. S11: Distribution of the asphericity of the Voronoi cell η as defined in eq. (4) in the zero-field case and for different field intensities (see legend). Arrows follow the progressive field-induced changes in the distribution.

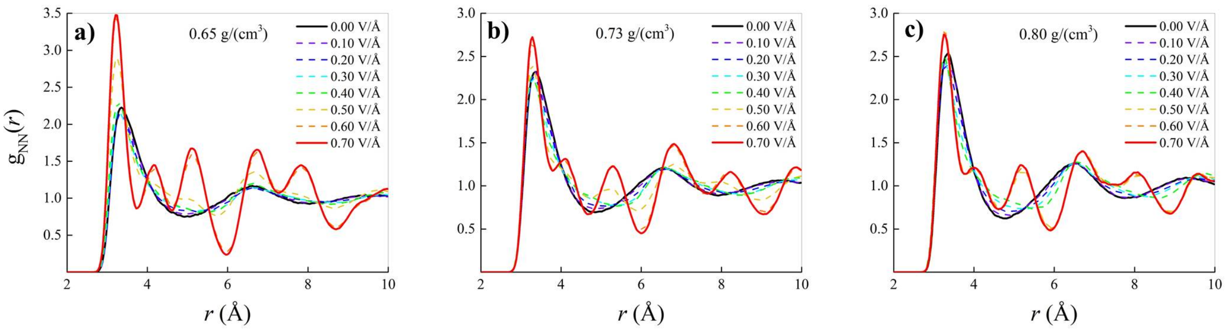

FIG. S12: Nitrogen-nitrogen radial distribution functions at different field strengths (see legends) calculated from the simulation boxes containing 256 molecules and reproducing densities equal to 0.65 g$\cdot$cm^{-3} (a), 0.73 g$\cdot$cm^{-3} (b), and 0.80 g$\cdot$cm^{-3} (c). Additionally to minor changes in the spacing and heights of the relative peaks, it can be observed that at higher densities the field threshold for electrofreezing lowers.

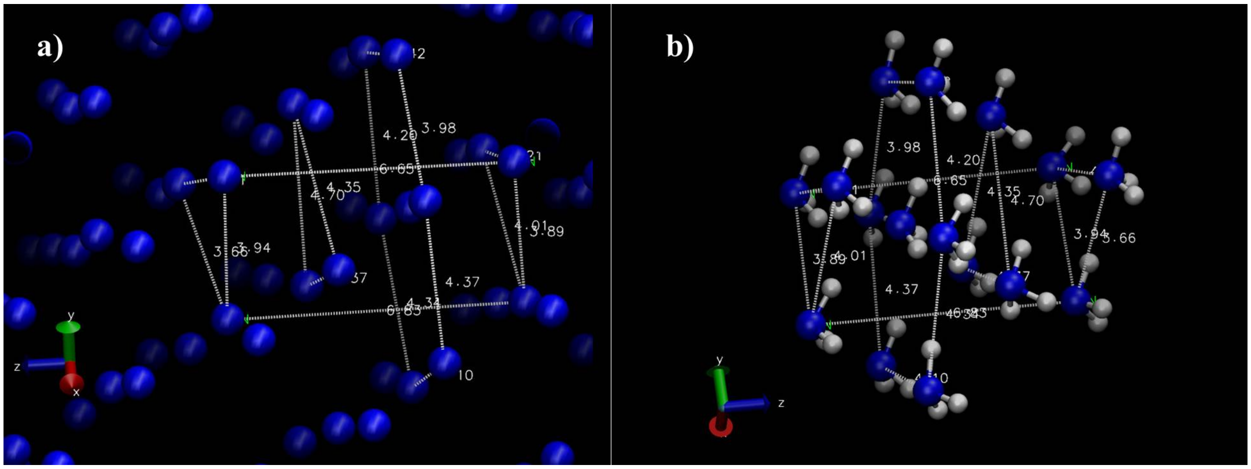

FIG. S13: (a) Typical *ABCABC* packing of a pseudo-*fcc* arrangement of the nitrogen sublattice observed in ammonia at intense field regimes (*i.e.*, $E > 0.50$ V/Å). (b) Visualization of the molecules identified in (a) also including the hydrogen atoms and removing the surrounding environment for clarity. It is worth noticing the orientation of the molecular axes along the field direction, which coincides with the blue axis of the reference system at the bottom-left of (b).

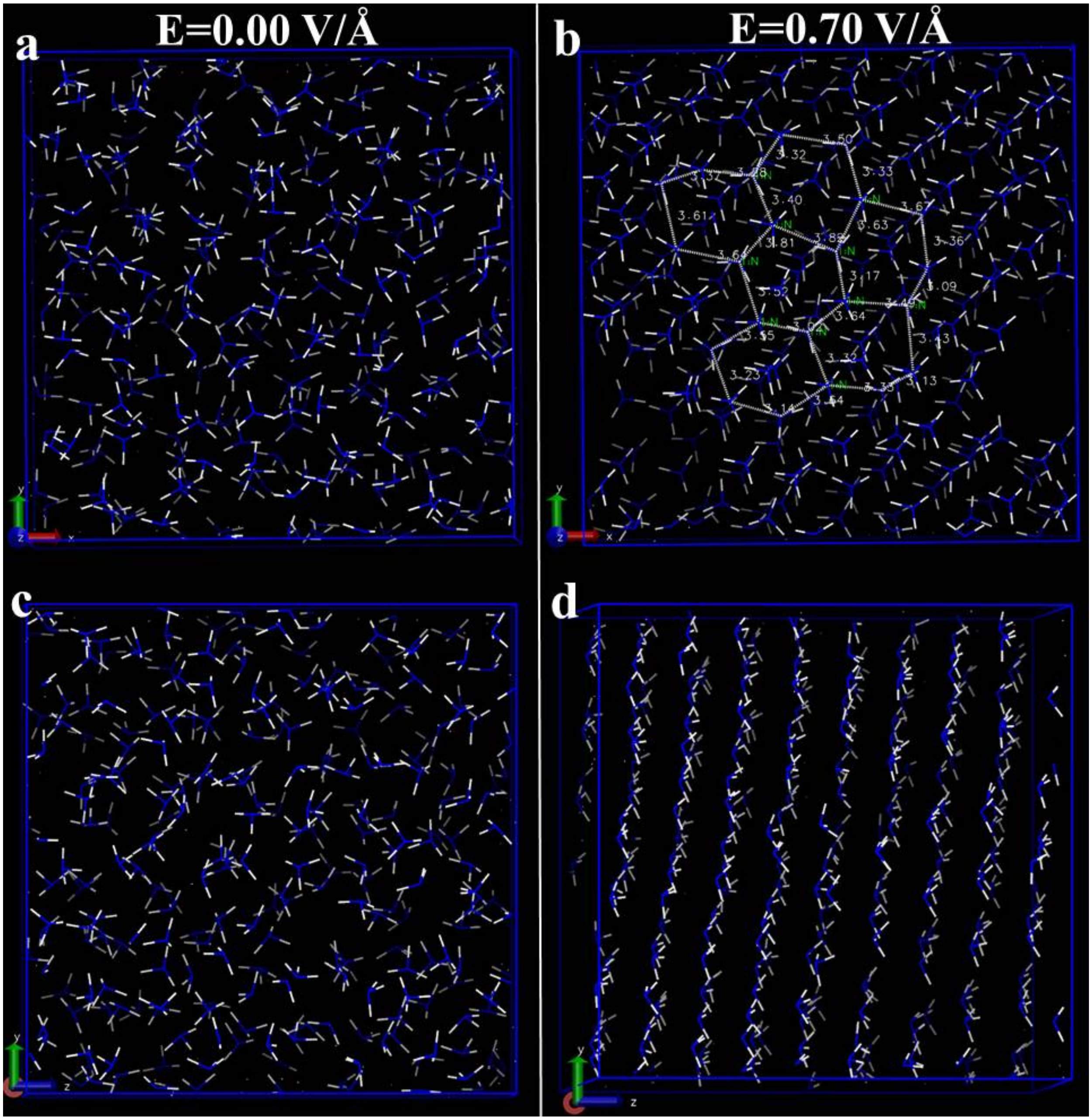

FIG. S14: (a/c) Snapshots, taken from different perspectives of the reference system (displayed at the bottom-left), of the simulation box at $\rho = 0.73$ g·cm^{-3} and containing 256 ammonia molecules in absence of the field. (b/d) Snapshots of the same sample when exposed to a field strength of 0.70 V/Å showing the formation of distorted hexagonal (chair-like) structures (b) and the stacking of the molecules in layers approximately perpendicular to the field direction (d), being this latter coincident with the blue axis of the reference system displayed at the bottom-left. It is worth noticing that in spite of the ferroelectric nature given by the field (*i.e.*, all molecules are oriented along the field axis), hydrogen atoms appear to be disordered.

[1] Kühne, T. D.; *et al.* CP2K: An electronic structure and molecular dynamics software package - Quickstep: Efficient and accurate electronic structure calculations. *J. Chem. Phys.* **2020**, *152*, 194103.

[2] Ceriotti, M.; Manolopoulos, D. E.; Parrinello, M. Accelerating the convergence of path integral dynamics with a generalized Langevin equation. *J. Chem. Phys.* **2011**, *134*, 084104.

[3] Krack, M. Pseudopotentials for H to Kr optimized for gradient-corrected exchange-correlation functionals. *Theor. Chem. Acc.* **2005**, *114*, 145.

[4] Becke, A. D. Density-functional exchange-energy approximation with correct asymptotic behavior. *Phys. Rev. A* **1988**, *38*, 3098; Lee, C.; Yang, W.; Parr, R. G. Development of the Colle-Salvetti correlation-energy formula into a functional of the electron density. *Phys. Rev. B* **1988**, *37*, 785.

[5] Grimme, S.; Antony, J.; Ehrlich, S.; Krieg, H. A consistent and accurate ab initio parametrization of density functional dispersion correction (DFT-D) for the 94 elements H-Pu. *J. Chem. Phys.* **2010**, *132*, 154104; Grimme, S.; Ehrlich, S.; Goerigk, L. Effect of the damping function in dispersion corrected density functional theory. *J. Comp. Chem.* **2011**, *32*, 1456.

[6] Boese, A. D.; Chandra, A.; Martin, J. M. L.; Marx, D. From ab initio quantum chemistry to molecular dynamics: The delicate case of hydrogen bonding in ammonia. *J. Chem. Phys.* **2003**, *119*, 5965.

[7] Bussi, G.; Donadio, D.; Parrinello, M. Canonical sampling through velocity rescaling. *J. Chem. Phys.* **2007**, *126*, 014101.

[8] Ceriotti, M.; Manolopoulos, D. E. Efficient First-principles calculation of the quantum kinetic energy and momentum distribution of nuclei. *Phys. Rev. Lett.* **2012**, *109*, 100604.

[9] Cassone, G. Nuclear quantum effects largely influence molecular dissociation and proton transfer in liquid water under an electric field. *J. Phys. Chem. Lett.* **2020**, *11*, 8983-8989.

[10] Markland, T. E.; Ceriotti, M. Nuclear quantum effects enter the mainstream. *Nature Reviews* **2018**, *2*, 0109.

[11] Resta, R. Macroscopic polarization in crystalline dielectrics: the geometric phase approach. *Rev. Mod. Phys.* **1994**, *66*, 899.

[12] Umari, P.; Pasquarello, A. Ab initio molecular dynamics in a finite homogeneous electric field.

Phys. Rev. Lett. **2002**, *89*, 157602.

[13] Brehm, M.; Kirchner, B. TRAVIS - A free analyzer and visualizer for Monte Carlo and molecular dynamics trajectories. *J. Chem. Inf. Model.* **2011**, *51*, 2007-2023.

[14] Thomas, M.; Brehm, M.; Fligg, R.; Voehringer, P.; Kirchner, B. Computing vibrational spectra from ab initio molecular dynamics. *Phys. Chem. Chem. Phys.* **2013**, *15*, 6608-6622.

[15] Marzari, N.; Vanderbilt, D. Maximally localized generalized Wannier functions for composite energy bands. *Phys. Rev. B* **1997**, *56*, 12847.

[16] Marzari, N.; Mostofi, A. A.; Yates, J. R.; Souza, I.; Vanderbilt, D. Maximally localized Wannier functions: Theory and applications. *Rev. Mod. Phys.* **2012**, *84*, 1419.

[17] Bromberg, A.; Kimel, S.; Ron, A. Infrared spectrum of liquid and crystalline ammonia. *Chem. Phys. Lett.* **1977**, *46*, 262-266.

[18] Marsalek, O.; Markland, T. E. Quantum dynamics and spectroscopy of ab initio liquid water: the interplay of nuclear and electronic quantum effects. *J. Phys. Chem. Lett.* **2017**, *8*, 1545-1551.

[19] Park, Y.; Kang, H.; Field, J. R.; Kang, H. The frequency-domain infrared spectrum of ammonia encodes changes in molecular dynamics caused by a DC electric field. *Proc. Natl. Acad. Sci. USA* **2019**, *116*, 23444-23447.

[20] Ojha, D.; Karhan, K.; Kühne, T. D. On the hydrogen bond strength and vibrational spectroscopy of liquid water. *Sci. Rep.* **2018**, *8*, 16888.

[21] Cassone, G.; Sponer, J.; Trusso, S.; Saija, F. Ab initio spectroscopy of water under electric fields. *Phys. Chem. Chem. Phys.* **2019**, *21*, 21205-21212.

[22] Luzar, A.; Chandler, D. Hydrogen-bond kinetics in liquid water. *Nature* **1996**, *379*, 55-57.

[23] Errington, J. R.; Debenedetti, P. G. Relationship between structural order and the anomalies of liquid water. *Nature* **2001**, *409*, 318-321.

[24] Steinhardt, P. J.; Nelson, D. R.; Ronchetti, M. Icosahedral Bond Orientational Order in Supercooled Liquids. *Phys. Rev. Lett.* **1981**, *47*, 1297.
